# Autonomous Toy Drone via Coresets for Pose Estimation

**DOI:** 10.3390/s20113042

**Published:** 2020-05-27

**Authors:** Soliman Nasser, Ibrahim Jubran, Dan Feldman

**Affiliations:** Robotics & Big Data Labs, University of Haifa, Haifa 3498838, Israel; snasser1@staff.haifa.ac.il (S.N.); dfeldman@univ.haifa.ac.il (D.F.)

**Keywords:** pose estimation, localization, indoor navigation and mapping, autonomous sensors for micro drones, coresets, caratheodory

## Abstract

A coreset of a dataset is a small weighted set, such that querying the coreset provably yields a (1+ε)-factor approximation to the original (full) dataset, for a given family of queries. This paper suggests accurate coresets (ε=0) that are subsets of the input for fundamental optimization problems. These coresets enabled us to implement a “Guardian Angel” system that computes pose-estimation in a rate >20 frames per second. It tracks a toy quadcopter which guides guests in a supermarket, hospital, mall, airport, and so on. We prove that any set of *n* matrices in $R^{d\times d}$ whose sum is a matrix *S* of rank *r*, has a coreset whose sum has the same left and right singular vectors as *S*, and consists of $O\left(dr\right)=O\left(d^{2}\right)$ matrices, independent of *n*. This implies the first (exact, weighted subset) coreset of $O(d^{2})$ points to problems such as linear regression, PCA/SVD, and Wahba’s problem, with corresponding streaming, dynamic, and distributed versions. Our main tool is a novel usage of the Caratheodory Theorem for coresets, an algorithm that computes its set in time that is linear in its cardinality. Extensive experimental results on both synthetic and real data, companion video of our system, and open code are provided.

## 1. Introduction and Motivation

Coresets is a powerful technique for data reduction that was originally used to improve the running time of algorithms in computational geometry (e.g., [1,2,3,4,5,6,7]). Later, coresets were designed for obtaining the first PTAS/LTAS (polynomial/linear time approximation schemes) for more classic and graph problems in theoretical computer science [8,9,10,11]. More recently, coresets appear in machine learning conferences [12,13,14,15,16,17,18,19,20,21] with robotics [12,13,15,16,18,20,21,22,23,24] and image [25,26,27] applications.

This paper has three goals:

(i) Introduce coresets to the robotics community and show how their theory can be applied in real-time systems and not only in the context of machine learning or theoretical computer science.
(ii) Suggest novel coresets for real-time kinematic systems, where the motivation is to improve the running time of an algorithm, by selecting a small subset of the moving points only once and then tracking and processing them (not the entire set) during the movement of the coreset in the next observed frames.
(iii) Provide a wireless and low-cost tracking system, IoTracker, that is based on mini-computers (“Internet of things”) that run coresets.

To obtain goal (i), we suggest a simple but powerful and generic coreset that approximates the center of mass of a set of points, using a sparse distribution on a small subset of the input points. While this “mean coreset” has many other applications, to obtain goal (ii) we use it to design a novel coreset for pose-estimation based on the alignment between two paired sets. We then show how this coreset enables us to compute the orientation of a rigid body, in particular a moving robot, which is a fundamental question in Simultaneous Localization And Mapping (SLAM) and computer vision; see references in [28].

For example, we prove that the result of running the classic Kabsch algorithm (which computes an optimal rotation between two sets of points) on the entire input, would yield the same result when applied on the coreset only. This holds even after the input set (including its coreset) is translated and rotated in space, without the need of recomputing the coreset. We prove that the coreset has constant size (independent of the number of input tracked points) for every given input set.

Although we proved the correctness of the coreset for the Kabsch algorithm, by its properties we expect that it would be useful for many other pose-estimation algorithms. As is common in coresets for system applications, even without this proof of correctness, the coreset may be used in practice for many other related pose estimation problems.

To demonstrate goal (iii), we install our tracking system in a university (and soon in a mall) and implement a “Guardian angel” application, that to our knowledge is the first implementation for fictional systems such as Skycall [29]: a safe and low-cost quadcopter leads a guest to its destination room based on preprogrammed routes and based on the walking speed of the human. The main challenge was to control a sensors-less quadcopter in a few dozens of frames per second using weak mini-computers. Unlike existing popular videos (e.g., [29]), in our video the quadcopter is autonomous in the sense that there is no hidden remote controller or another human in the loop, see [30].

**Figure 1:**
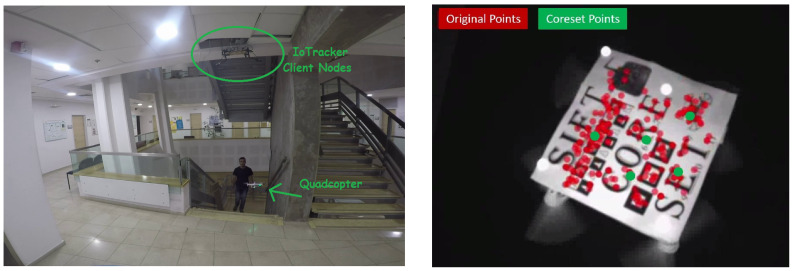
Snapshots from the **companion video** in: [30]. (**Left**) “Guardian Angle” system. A safe and low-cost quadcopter autonomously leads a guest to its destination (**Right**) “real-time computation of the Kabsch-Coreset. A set *P* of |P|=n interest points detected on a planar pattern placed on a drone (red points). A subset (coreset) C⊆P of |C|=5 points (green points). It is guaranteed that the pose of the drone when computed using the weighted coreset *C* will be the same as the pose computed from the whole set *P*.

This “Guardian angel” was our main motivation and inspiration for designing the coreset in this paper; see Figure 1.

We note that our paper is not about suggesting the best algorithm for pose-estimation, the best tracking system, or about localization of quadcopters. As stated above, our goals are to show the process cycle from deep theorems in computational geometry, as the Caratehorody Theorem, to real-time and practical systems that use coresets. Nevertheless, we are not aware of similar coresets for pose estimation of kinematic data or low-cost wireless tracking systems that can be used for hovering of a very unstable quadcopter in dozens of frames per second.

## 2. Related Work and Comparison

In this section we discuss related work on the general Pose Estimation problem and related solutions such as Prespective-n-Point (PnP), Iterative Closest Point (ICP), and other approaches to solve it. Finally, we suggest how these algorithms can be applied on the coresets of this paper and conclude with summary on related coreset constructions.

**Pose Estimation.** The pose estimation problem is also called the alignment problem, since given two paired point sets, *P* and *Q*, the task is to find the Euclidean motion that brings *P* into the best possible alignment with *Q*. We focus on the case where this alignment is the translation $\mu^{*}$ and rotation *R* of *P* that minimizes the sum of squared distances to the point of *Q*. For |P|=|Q|=n points in $R^{d}$, the optimal translation $\mu^{*}$ is simply the mean of *Q* minus the means of *P*, each can be computed in O(nd) time. Computing the optimal rotation $R^{*}$ (Wahba’s Problem [31]) can be computed independently via the Kabsch algorithm [32] in $O(nd^{2})$ time; see Theorem 2.

**In the PnP Problem.** We are given a set of (known) 3D points and a set of *n* 2D points (observed points). If we have the camera’s internal parameters, a set of *n* lines in 3D space can be computed from the 2D set of points. The goal is to align the set of 3D points with the set of 3D lines, which makes the problem hard unlike the problems that are discussed in this paper. Indeed, exact solutions for the PnP problem are known only for the case n≤4, and no provable approximations are known when the data is noisy and n>4, even for the case of sum of squared distances. The Kabsch Coreset in this paper may be used to improve the running time of common PnP heuristics by running them on the coreset. Unlike their usage for Kabsch Algorithm, the theoretical guarantees of the coreset would no longer hold.

A sort of coreset of 4 point for PnP was suggested in [33]. However, unlike our Kabsch coreset, this set is not a subset of the input and provides no optimality guarantees.

**ICP.** In the previous paragraphs we assumed that the matching between *P* and *Q* is given. The standard and popular solution for solving the matching and pose-estimation problems is called Iterative Closest Point (ICP) proposed by Besl and McKay [34]; see [35] and references therein. This algorithm starts with a random matching (mapping) between *P* and *Q*, then: (a) runs the Kabsch algorithm on this pair of sets, and (b) rematches each point in *P* to its nearest point in *Q*, then returns to step (a). Variations and speed-ups can be found in [36,37,38].

**Faster and Robust Matching Using Coresets.** Our Kabsch coreset, similarly to the Kabsch algorithm, assumes that the matching between the points in the registered and observed frame is given. Matching is a much harder problem than, e.g., the Kabsch algorithm (that can be solved in O(n) time) in the sense that we have n! permutations. Nevertheless, the mean coreset that we will present can reduce the running time and increase the robustness of the matching process.

For example, in ICP, each point in $P\subseteq R^{3}$ is assigned to its nearest neighbour (NN) in *Q* which take O(|P|·|Q|) time. Using our Kabsch coreset for *P*, the running time of the algorithm reduces to O(|Q|). This also implies that NN matching can be replaced in existing applications by a slower but better algorithm (e.g., cost-flow [39]) that will run on the small coreset. This will improve the matching step of ICP, without increasing the existing running times. Such an improvement is relevant even for nonkinematic (single) pair *P* and *Q* of points.

Table 1 concludes the time complexity comparison of solving each step of the localization problem with/without using our coresets. The first row of the table represents the case where the matching has already been computed, and what is left to compute is the optimal rotation between the two sets of points. The second row represents step (b) of the localization problem, where the matching needs to be computed given the rotation. In this case, a perfect matching between a set of size *k* to a set of size *m* can be achieved, according to [40], in $O(\sqrt{m+k}mk\log\left(m+k\right)$ time. Without using a coreset, the size of both sets is *n*. When using a coreset, the size of *P* is reduced to rd, although the size of *Q* remains *n*. The last row of Table 1 represents a case where we need to compute the matching between two sets of points and the correct alignment is not given. In this case there are n! possible permutations of the original set, each with its own optimal rotation. Using the coreset, the number of permutations reduces to roughly (rd)! since it suffices to match correctly only the coreset points.

**Relation to Other Coresets.** A long line of research is dedicated to the problem of approximating ${∥Ax∥}^{2}=x^{T}\left(\sum_{i=1}^{n}a_{i}a_{i}^{T}\right)x$ for every $x\in R^{d}$, which is related to SVD and linear regression as explained in Section 3.1. A breakthrough with applications to graph sparsification was suggested in [41], via a deterministic coreset construction (weighted subset) of size $O(d/\varepsilon^{2})$ that yields a (1+ε) approximation for $∥Ax∥$. This result was generalized to low *k*-rank approximation problem (*k*-SVD, or *k*-PCA) using $O(k/\varepsilon^{2})$ samples in [42], and for Frobenius norm using $O(k^{2}/\varepsilon^{2})$ samples in [12].

If the coreset is restricted only to be a weighted subset of $R^{d}$, and not from the input set, then its cardinality can be reduced to O(k/ε) points by [43]. More properties may be obtained for approximating other problems (such as *k*-means) using O(k/ε) points using [44,45].

However, it is not clear how the approximation error will affect the output rotation matrix that is returned by the Kabsch algorithm via the above coreset. In this paper we focus on exact coresets that have no approximation error ε. This allows us to obtain the optimal solution for the problem. Since our mean coreset does not introduce any error, it can be used in any applications that aim to compute any functions $f\left(AA^{T}\right)=f\left(\sum_{i}a_{i}a_{i}^{T}\right)$, since it preserves the sum $\sum_{i}a_{i}a_{i}^{T}$.

The only such accurate coreset that we know is $S\in R^{d\times d}$ for a matrix A=USx, where $U\in R^{n\times d}$ is an arbitrary orthonormal base of the columns of *A* (e.g., using the SVD $A=UDV^{T}$ or U=Q from the QR decomposition (Gram–Schmidt) A=QR of *A*). Hence, ${∥Ax∥}_{2}=∥Sx∥$ for every $x\in R^{d}$ and there is no approximation error. However, in this case the rows of *S* are not a scaled subset of the input rows. Besides numerical and interpretation issues, we cannot use this coreset *S* for kinematic data since we do not have a subset of points to track over time or between frames.

Coreset for sum of 1-rank positive definite matrices of size $O(d/\varepsilon^{2})$ were described, e.g., in [46]; see references therein. Our mean coreset is larger but implies such an exact result and is more general (sum of any d×d matrices).

## 3. Warm Up: Mean Coreset

Given a set *P* of *n* points (*d*-dimensional vectors), our basic suggested tool is a small weighted subset of *P*, that we call *mean coreset*, whose weighted mean is exactly the same as the mean of the original set. In general, we can simply take the mean of *P* as a coreset of size 1. However, we require that the coreset will be a subset of the input set *P*. Moreover, we require that the vector of the multiplicative weights will be a sparse distribution over *P*, i.e., a positive vector with an average entry of 1. There are at least three reasons for using this coreset definition in practice, especially for real-time kinematic/tracking systems:

*(i)*  **Numerical stability:** Every *d* linearly independent points in *P* span their mean. However, this coreset yields huge positive and negative coefficients that canceled each other and resulted in high numerical error. Our requirement that the coreset weights will have positive weights whose average is 1 makes these phenomena disappear in practice.
*(ii)*  **Efficiency:** A small coreset allows us to compute the mean of a kinematic (moving) set of points faster, by computing the mean of the small coreset in each frame, instead of the complete set of points. This also reduces the time and probability of failure of other tasks such as matching points between frames. This is explained in Section 1.
*(iii)*  **Kinematic Tracking:** In the next sections we track the orientation of an object (robot or a set of vectors) by tracking a kinematic representative set (coreset) of markers during many frames. This coreset is computed once for the many following frames. Such tracking is impossible when the coreset is not a subset of the tracked points.

We now formally define this mean coreset.

**Definition** **1** (Mean coreset)**.** *A* distribution vector $u=(u_{1},\cdots ,u_{n})$ *is a vector whose entries are non-negative and sum to one. A* weighted set *is a pair* (P,u) *where* $P=\left\{p_{1},\cdots ,p_{n}\right\}$ *is an ordered set in* $R^{d}$*, and u is a distribution vector of length* |P|*.*

*A weighted set*(S,w)*is a* mean coreset *for the weighted set* (P,u) *if* S⊆P *and their weighted mean is the same, i.e.,*

$$\sum_{i=1}^{n}u_{i}p_{i}=\sum_{j=1}^{\left|S\right|}w_{j}s_{j},$$

*where* $S=\left\{s_{1},\cdots ,s_{\left|S\right|}\right\}$*. The* cardinality *of the mean coreset* (S,w) *is* |S|*.*

Of course *P* is a trivial coreset of *P*. However, the coreset *S* is efficient if its size $\left|S\right|=|\left\{i|w_{i}>0\right\}|$ is much smaller than |P|=n. This is related to the Caratheodory Theorem [47] from computational geometry, that states that any convex combination of a set *P* of points (in particular, its mean) is a convex combination of at most d+1 points in *P*.

We first suggest an inefficient construction in Algorithm 1 to obtain a mean coreset of only d+1 points, i.e., independent of *n*, for a set of *n* points. This is based on the proof of the Caratheodory Theorem which we give for completeness and takes $O(n^{2}d^{2})$ time, which is impractical for the applications in this paper.

**Figure 2:**
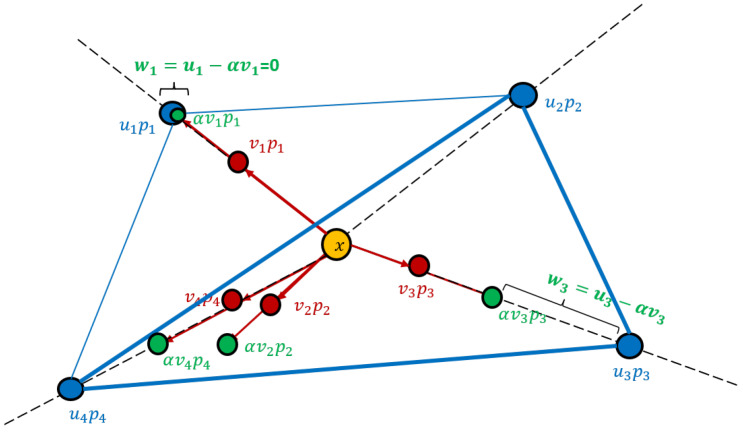
A weighted set (P,u) whose weighted mean is $\sum_{i=1}^{4}u_{i}p_{i}=0$ corresponds to four points (in blue) whose sum is the origin (in orange). Algorithm 1 first computes a weighted set (P,v) (red points) whose weighted mean is the origin, and sum of weights is $\sum_{i=1}^{4}v_{i}=0$. The weights are scaled by α>0 until $\alpha v_{i}=u_{i}$ for some *i* (i=1 in the figure). The resulting weighted set (P,αv) in green is subtracted from the input (P,u) to obtain (P,u−αv)=(P,w), where $w_{1}=0$ so $p_{1}$ can be removed. Algorithm 1 then continues iteratively with the remaining points until (P,w) has |P|=d+1 weighted points.

**Overview of Algorithm 1 and its correctness.** The input is a weighted set (P,u) whose points are denoted by $P=\left\{p_{1},\cdots ,p_{n}\right\}$; see Figure 2 for an illustration. We assume n>d+1, otherwise (S,w)=(P,u) is the desired coreset. Hence, the n−1>d points $p_{2}-p_{1}$, $p_{3}-p_{1},p_{4}-p_{1},\ldots$ must be linearly dependent. This implies that there are reals $v_{2},\cdots ,v_{n}$, which are not all zeros, such that

(1) $$\sum_{i=2}^{n}v_{i}\left(p_{i}-p_{1}\right)=0.$$

These reals are computed in Line 6 by solving the system of linear equations. This step dominates the running time of the algorithm and takes $O(nd^{2})$ time using, e.g., SVD. The definition

(2) $$v_{1}=-\sum_{i=2}^{n}v_{i}$$

in Line 7, guarantees that

(3) $$v_{j}<0\;\mathrm{for}\;\mathrm{some}\;j\in \left[n\right],$$

and that

(4) $$\begin{array}{cc} \sum_{i=1}^{n}v_{i}p_{i} & =v_{1}p_{1}+\sum_{i=2}^{n}v_{i}p_{i}=\left(-\sum_{i=2}^{n}v_{i}\right)p_{1}+\sum_{i=2}^{n}v_{i}p_{i}\\ & =\sum_{i=2}^{n}v_{i}\left(p_{i}-p_{1}\right)=0, \end{array}$$

where the second equality is by (2), and the last is by (1). Hence, for every α∈R, the weighted mean of *P* is

(5) $$\sum_{i=1}^{n}u_{i}p_{i}=\sum_{i=1}^{n}u_{i}p_{i}-\alpha \sum_{i=1}^{n}v_{i}p_{i}=\sum_{i=1}^{n}\left(u_{i}-\alpha v_{i}\right)p_{i},$$

where the first equality holds since $\sum_{i=1}^{n}v_{i}p_{i}=0$ by (4). The definition of α in Line 8 guarantees that $\alpha v_{i^{*}}=u_{i^{*}}$ for some $i^{*}\in \left[n\right]$ and that $u_{i}-\alpha v_{i}\geq 0$ for every i∈[n]. Hence, the set *S* that is defined in Line 10 contains at most n−1 points, and its set of weights $\left\{u_{i}-\alpha v_{i}\right\}$ is non-negative. Notice that if α=0, we have that $w_{k}=u_{k}>0$ for some k∈[n]. Otherwise, by (3), there is j∈[n] such that $w_{j}=u_{j}-\alpha v_{j}>0$. Hence, |S|≠∅. The sum of the positive weights is thus the total sum of weights,

$$\sum_{p_{i}\in S}^{n}w_{i}=\sum_{i=1}^{n}\left(u_{i}-\alpha v_{i}\right)=\sum_{i=1}^{n}u_{i}-\alpha \cdot \sum_{i=1}^{n}v_{i}=1,$$

where the last equality holds by (2) and since *u* is a distribution vector. This and (5) proves that *S* is a mean coreset as in Definition 1 of size n−1. In Line 12 we repeat this process recursively until there are at most d+1 points left in *S*. For O(n) iterations the overall time is thus $O(n^{2}d^{2})$.

The correctness of the following lemma follows mainly by the Caratheodory Theorem [47] from computational geometry.

**Lemma** **1.** *Let* $P=\left\{p_{1},\cdots ,p_{n}\right\}\subseteq R^{d}$ *be a set of* n>d+1 *points and* $u=\left\{u_{1},\cdots ,u_{n}\right\}$ *be a distribution. Let* (S,w) *be the output of a call to* MEAN-CORESET(P,u) *; see Algorithm 1. Then* (S,w) *is a mean coreset of* (P,u) *. This takes* $O(n^{2}d^{2})$ *time.*

We then use the fact that our mean coresets are composable [48,49,50,51]: a union of coresets can be merged and reduced again recursively. To reduce the running time of Algorithm 1, we run it only on the first d+2 points of *P* and reduce the d+2 points to a coreset of d+1 points in $O(d^{3})$ time using a single iteration. We then add a new point to the previously compressed d+1 points, compress again, then repeat for each of the remaining points using n=d+1 in Lemma 1 for every point update.

**Overview of Algorithm 2 and its correctness.** We denote $\left[n\right]=\left\{1,\cdots ,n\right\}$ for every integer n≥1. In Lines 1–3 we respectively set n=d+1, initialize *S* with the first d+1 points from stream, and set the weight of all the points in *S* to be $\frac{1}{d+1}$. In Line 4 we begin to read the points in the (possibly infinite) input stream of points. In Line 5 we update this counter *n*, and in Line 6 we read the *n*th point from the stream. The set *P* in Line 7 is the union of the coreset for the points read until now with the new *n*th point *p*.

In Line 8 we define a distribution vector *u* such that the weighted set (P,u) has the same mean as the mean of the *n* points $p_{1},\cdots ,p_{n}$ that were read until now. The intuition is that the new points represent a fraction of 1/n from the *n* points seen so far, but *S* (the rest of points in *P*) represents (n−1)/n input points. If the *i*th point in *S* has a weight $w_{i}$, it means that it represents a fraction of $w_{i}$ from *S*, i.e., fraction of $w_{i}\left(n-1\right)/n$ from all the data. Indeed, the mean of the *n* read points $p_{1},\cdots ,p_{n}$ and *P* is the same,

(6) $$\begin{array}{cc} \frac{1}{n}\sum_{i=1}^{n}p_{i} & =\frac{1}{n}\sum_{i=1}^{n-1}p_{i}+\frac{p_{n}}{n}=\frac{n-1}{n}\sum_{i=1}^{\left|S\right|}w_{i}s_{i}+\frac{p_{n}}{n}\\ & =\sum_{i=1}^{\left|S\right|}u_{i}p_{i}+\frac{p_{n}}{n}=\sum_{i=1}^{\left|P\right|}u_{i}p_{i}, \end{array}$$

where the second equality holds since $\frac{1}{n-1}\sum_{i=1}^{n-1}p_{i}=\sum_{i=1}^{\left|S\right|}w_{i}s_{i}$, and the last equality holds since $p_{n}=p_{\left|P\right|}$. Moreover, *u* is a distribution vector since

$$\sum_{i=1}^{n}u_{i}=\frac{1}{n}+\sum_{i=1}^{\left|S\right|}\frac{w_{i}\left(n-1\right)}{n}=\frac{1}{n}+\frac{n-1}{n}=1,$$

where the second equality is since *w* is a distribution vector by induction.

In Line 9, we compute a mean coreset (S,w) for (P,u). Since |P|=d+2, by Lemma 1 this takes $O(d^{3})$, and by (6) (S,w) is also the mean coreset for the *n* points read until now. In Line 10 we output (S,w) and repeat for the next point. The required memory is dominated by the set *P* of d+2 points. We conclude with the following theorem.

**Theorem** **1.** *Let* stream *be a procedure that outputs a new point in* $R^{d}$ *after each call. A call to* STREAMING-CORESET(stream) *outputs a mean coreset of cardinality* d+1 *for the first n points in* stream *, for every* n≥1 *. This takes* $O(d^{3})$ *time for each point update, overall of* $O(nd^{3})$ *time and using at most* d+2 *points in memory.*

 **Algorithm 1:**
MEAN-CORESET(P,u)
 
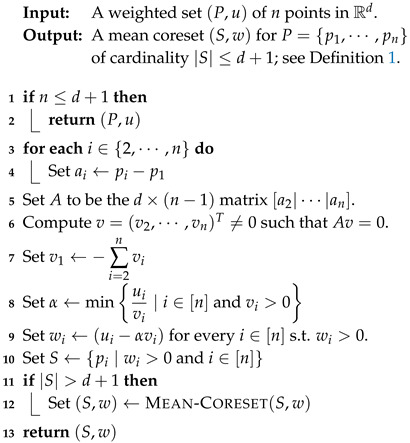


 **Algorithm 2:**
STREAMING-CORESET(stream)
 
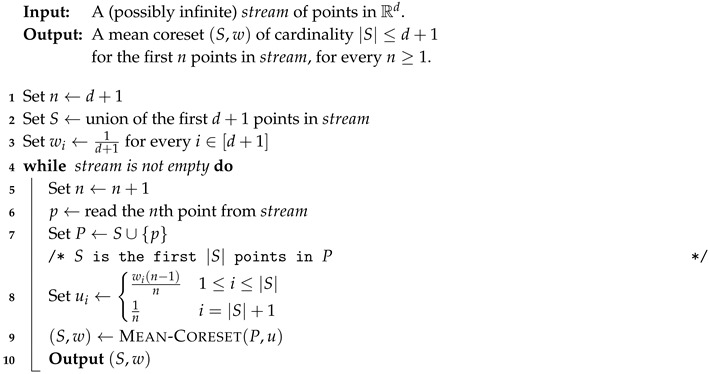


### 3.1. Example Applications

**Coreset for 1-mean queries.** A coreset for *k*-mean queries of a set $P\subseteq R^{d}$ approximates the sum of squared distances over the points of *P* to any given set of *k* centers (points in $R^{d}$). There is a long line of research for this type of coresets [11,52,53,54,55,56,57]. Algorithm 2 yields the first accurate coreset (no approximation error) weighted subset S⊆P of size d+3 for the case k=1. The solution also holds for streaming data (and distributed/dynamic data as explained below).

**Corollary** **1.** *Let* $P^{\prime }=\left\{p_{1}^{\prime },\cdots ,p_{n}^{\prime }\right\}$ *be a set of points in* $R^{d}$ *. Let* stream *be a corresponding stream whose ith point is* $\left(p^{\prime }|{∥p^{\prime }∥}^{2}|1\right)\in R^{d+2}$ *for every* i≥1 *. Let* (S,w) *be the nth outputted pair of a call to* STREAMING-CORESET(stream) *, and* $S^{\prime }=\left\{p^{\prime }\in P^{\prime }|p\in S\right\}\subseteq P^{\prime }$ *. Then* |S|=d+3 *and for every* $x\in R^{d}$ *we have*

$$\sum_{i=1}^{n}{∥p_{i}^{\prime }-x∥}^{2}=\sum_{i=1}^{d+3}w_{i}{∥s_{i}-x∥}^{2}.$$

**Proof.**  Simple calculations show that

$$\begin{array}{cc} \sum_{i=1}^{n}{∥p_{i}^{\prime }-x∥}^{2} & =\sum_{i=1}^{n}{∥p_{i}^{\prime }∥}^{2}+n{∥x∥}^{2}-2x^{T}\sum_{i=1}^{n}p_{i}^{\prime }=\left(\sum_{i=1}^{n}{∥p_{i}^{\prime }∥}^{2},n,\sum_{i=1}^{n}p_{i}^{\prime }\right)\left(\begin{array}{c} 1\\ {∥x∥}^{2}\\ -2x^{T} \end{array}\right)\\ & =\left(\sum_{i=1}^{n}\left({∥p_{i}^{\prime }∥}^{2},1,p_{i}^{\prime }\right)\right)\left(\begin{array}{c} 1\\ {∥x∥}^{2}\\ -2x^{T} \end{array}\right)=\left(\sum_{i=1}^{d+3}w_{i}s_{i}\right)\left(\begin{array}{c} 1\\ {∥x∥}^{2}\\ -2x^{T} \end{array}\right)\\ & =\left(\sum_{i=1}^{d+3}w_{i}{∥p_{i}^{\prime }∥}^{2}|\sum_{i=1}^{d+3}w_{i}|\sum_{i=1}^{d+3}w_{i}p_{i}^{\prime }\right)\left(\begin{array}{c} 1\\ {∥x∥}^{2}\\ -2x^{T} \end{array}\right)=\sum_{i=1}^{d+3}w_{i}{∥s_{i}-x∥}^{2}. \end{array}$$

 □

**Sum coreset for matrices.** Theorem 1 implies that we can compute the sum of *n* matrices in $R^{d\times d}$ using a weighted subset of $d^{2}$ matrices, simply by concatenating the entries of each matrix to a vector in $R^{d^{2}}$. In Section 4 we reduce this size for the case where we are only interested in the left+right singular vectors of the matrix. This reduction is theoretically small but allowed us to reduce the number of required markers on the object tracked by our system by more than half in the third paragraph of Section 6.2, which was critical to the IR tracking version of our system.

**Coreset for SVD.** Let $A\in R^{n\times d}$. Our mean coreset implies that there is a matrix *S* that consists of $O(d^{2})$ scaled rows in *A* such that for every $x\in R^{d}$, ${∥Ax∥}_{2}={∥Sx∥}_{2}.$ This is since

$${∥Ax∥}_{2}^{2}={\left(Ax\right)}^{T}\left(Ax\right)=x^{T}A^{T}Ax=x^{T}\left(\sum_{i=1}^{n}a_{i}a_{i}^{T}\right)x.$$

The rightmost term can be computed using a mean coreset for matrices as defined above.

**Coreset for Linear Regression.** In the case of linear regression, we are also given a vector $b\in R^{n}$ and wish to compute a matrix *S* of $O(d^{2})$ weighted rows from *A* and a vector *v* of the same size, such that for every $x\in R^{d}$ we have

$${∥Ax-b∥}_{2}={∥Sx-v∥}_{2}.$$

This can be obtained by replacing *A* with [A∣−b] in the previous example.

**Streaming, Distributed and Dynamic computation.** Theorem 1 implies that we can compute the above coresets also for possibly infinite streaming set of row vectors or matrices. Similarly, using *m* machines the (parallel) running time can be reduced by a factor of *m* by sending the *i*th point in the stream to the (imodm) th machine [58] using communication of $O(d^{2})$ points of the final coreset to a main machine. Unlike the common usage of binary merge-reduce trees (e.g., [51]), the approximation error, memory, and time do not increase with *n*, for unbounded stream, due to the fact that the coresets are exact.

Such composable coresets support deletion/insertion of a point in logarithmic update time (but linear space) in the number *n* of existing points in the set; see details in [51].

## 4. Application for Kinematic Data: Kabsch Coreset

To track a kinematic set of points (e.g., markers or visual features on a rigid body), we define its initial (zero) positions $\left\{p_{1},\cdots ,p_{n}\right\}$ as the set of *n* rows of a matrix $P\in R^{n\times 3}$ which is centered around the origin and compare it to the observed set, i.e., rows $\left\{q_{1},\cdots ,q_{n}\right\}$ of a matrix $Q\in R^{n\times 3}$ in the current time or frame. The difference (translation and rotation) between *P* and *Q* tells us the current position of the set. Using the Maximum Likelihood approach and the common assumption of Gaussian noise (which has physical justification), the optimal solution is the translation and rotation of *P* that minimize the sum of squared distances to the corresponding points (rows) in *Q*. Consider the problem of computing the rotation matrix that minimizes the sum of squared distances between the corresponding sets,

$$\mathrm{cost}\left(P,Q,R\right):=\sum_{i=1}^{n}{∥p_{i}-q_{i}R∥}^{2},$$

This is known as Wahba’s Problem [31]. We denote this minimum by

$$\mathrm{OPT}\left(P,Q\right):=\min_{R}\mathrm{cost}\left(P,Q,R\right)=\mathrm{cost}\left(P,Q,R^{*}\right),$$

where the minimum is over every rotation matrix $R\in R^{d\times d}$.

**Tracking translation.** Consider the problem of computing the optimal translation, i.e., the translation vector $t^{*}\in R^{d}$ that minimizes

$$\mathrm{cost}\left(P,Q,t\right):=\sum_{i=1}^{n}{∥p_{i}-q_{i}-t∥}^{2},$$

over every $t\in R^{d}$. Easy calculations show that the optimal translation is the mean (center of mass) of *Q*. This mean can be maintained by tracking only the small mean coreset of *Q* over time as defined in Section 3, even without knowing the matching between between the points in *P* and *Q*.

In this section we thus focus on the more challenging problem of computing the rotation *R* that minimizes the sum of squared distances between the points of *P* and QR.

The Kabsch algorithm [32] suggests the following simple but provably optimal solution for Wahba’s problem. Let $UDV^{T}$ be a Singular Value Decomposition (SVD) of the matrix $P^{T}Q$. That is, $UDV^{T}=P^{T}Q$, $U^{T}U=V^{T}V=I$, and $D\in R^{d\times d}$ is a diagonal matrix whose entries are nonincreasing. In addition, assume that det(U)det(V)=1, otherwise invert the signs of one of the columns of *V*. Note that *D* is unique but there might be more than one such factorization.

**Theorem** **2** ([32])**.** *The matrix* $R^{*}=VU^{T}$ *minimizes* cost(P,Q,R) *over every rotation matrix R, i.e.,* $\mathrm{OPT}\left(P,Q\right)=\mathrm{cost}\left(P,Q,R^{*}\right).$

We now suggest a coreset (sparse distribution) for this problem.

**Definition** **2** (Kabsch Coreset)**.** *Let*$w\in S^{n}$*be a distribution. Let*$\tilde{P},\tilde{Q}\in R^{n\times d}$*denote the matrices whose ith row is*$\sqrt{w_{i}}p_{i}$*and*$\sqrt{w_{i}}q_{i}$*, respectively, for every*$i\in \left\{1,\cdots ,n\right\}$*. Then w is a* Kabsch coreset *for the pair* (P,Q) *if for every pair of rotation matrices* $A,B\in R^{d\times d}$ *and every pair of vectors* $\mu ,\nu \in R^{d}$ *the following holds: A rotation matrix* $\tilde{R}$ *that minimizes* $\mathrm{cost}(\tilde{P}A+\mu ,\tilde{Q}B+\nu ,R)$ *over every rotation matrix R, is also optimal for* (PA+μ,QB+ν)*, i.e.,*

$$\mathrm{OPT}\left(PA+1^{T}\mu ,QB+1^{T}\nu \right)=\mathrm{cost}\left(PA+1^{T}\mu ,QB+1^{T}\nu ,\tilde{R}\right),$$

*where* $1^{T}=\left(1,\cdots ,1\right)\in R^{n}$*.*

This implies that we can use the same coreset even if the set *Q* is translated or rotated over time. Such a coreset is efficient if it is also small (i.e., the distribution vector *w* is sparse).

Recall that $UDV^{T}$ is the SVD of $P^{T}Q$, and let *r* denote the rank of $P^{T}Q$, i.e., number of nonzero entries in the diagonal of *D*. Let $D_{r}\in R^{d\times d}$ denote the diagonal matrix whose diagonal is 1 in its first *r* entries, and 0 otherwise.

**Lemma** **2.** *Let* $R=GF^{T}$ *be a rotation matrix, such that F and G are orthogonal matrices, and* $GD_{r}F^{T}=VD_{r}U^{T}$ *. then R is an optimal rotation, i.e.,*

OPT(P,Q)=cost(P,Q,R).

*Moreover, the matrix* $VD_{r}U^{T}$ *is unique and independent of the chosen Singular Value Decomposition* $UDV^{T}$ *of* $P^{T}Q$ *.*

**Proof.**  It is easy to prove that *R* is optimal, if

(7) $$\mathrm{Tr}\left(RP^{T}Q\right)=\mathrm{Tr}\left(D\right);$$

see [59] for details. Indeed, the trace of the matrix $RP^{T}Q$ is

$$\begin{array}{cccc} \mathrm{Tr}(RP^{T}Q) & = & \mathrm{Tr}\left(RUDV^{T}\right)=\mathrm{Tr}\left(GF^{T}\left(UDV^{T}\right)\right) & \\ & = & \mathrm{Tr}(GD_{r}F^{T}\cdot UDV^{T}) & \left(8\right)\\ & & +\mathrm{Tr}(G\left(I-D_{r}\right)F^{T}\cdot UDV^{T}). & \left(9\right) \end{array}$$

Term (8) equals

(10) $$\begin{array}{c} \mathrm{Tr}\left(GD_{r}F^{T}\cdot UDV^{T}\right)=\mathrm{Tr}\left(VD_{r}U^{T}\cdot UDV^{T}\right)\\ =\mathrm{Tr}\left(VDV^{T}\right))=\mathrm{Tr}\left(DV^{T}V\right)=\mathrm{Tr}\left(D\right), \end{array}$$

where the last equality holds since the trace is invariant under cyclic permutations. Term (9) equals

(11) $$\begin{array}{c} \mathrm{Tr}(G\left(I-D_{r}\right)F^{T}\cdot UDV^{T})\\ =\mathrm{Tr}(G\left(I-D_{r}\right)F^{T}\cdot {\left(D_{r}U^{T}\right)}^{T}DV^{T})\\ =\mathrm{Tr}(G\left(I-D_{r}\right)F^{T}\cdot {\left(V^{T}GD_{r}F^{T}\right)}^{T}DV^{T})\\ =\mathrm{Tr}(G\left(I-D_{r}\right)F^{T}\cdot FD_{r}^{T}G^{T}V\cdot DV^{T})\\ =\mathrm{Tr}(G\cdot \left(I-D_{r}\right)D_{r}\cdot G^{T}V\cdot DV^{T})=0, \end{array}$$

where the last equality follows since the matrix $\left(I-D_{r}\right)D_{r}$ has only zero entries. Plugging the last equality and (10) in (8) yields $\mathrm{Tr}\left(RP^{T}Q\right)=\mathrm{Tr}\left(D\right)$. Using this and (7) we have that *R* is optimal.

For the uniqueness of the matrix $VD_{r}U^{T}$, observe that for $N=P^{T}Q=UDV^{T}$ we have

(12) $${\left(N^{T}N\right)}^{1/2}{\left(N\right)}^{+}=\left(VDV^{T}\right)\left(VD^{+}U^{T}\right)=VD_{r}U^{T}.$$

Here, a squared root $X^{1/2}$ for a matrix *X* is a matrix such that ${\left(X^{1/2}\right)}^{2}=X$, and $X^{+}$ denote the pseudo inverse of *X*. Let $FEG^{T}$ be an SVD of *N*. Similarly to (12), ${\left(N^{T}N\right)}^{1/2}{\left(N\right)}^{+}=GD_{r}F^{T}$.

Since $N^{T}N=VD^{2}V^{T}$ is a positive-semidefinite matrix, it has a unique square root. Since the pseudo inverse of a matrix is also unique, we conclude that ${\left(N^{T}N\right)}^{1/2}{\left(N\right)}^{+}$ is unique, and thus $VD_{r}U^{T}=GD_{r}F^{T}$. □

**Overview of Algorithm 3.** The input is a pair (P,Q) of n×d matrices that represent two paired set of points in $R^{d}$. To obtain an object’s pose, we need to apply the Kabsch algorithm on the matrix $P^{T}Q=\sum_{i}p_{i}^{T}q_{i}$; see Theorem 2. Algorithm 3 outputs a sparse weight vector $w=(w_{1},\cdots ,w_{n})$ such that the summation $P^{T}Q$ equals to the weighted sum $\sum_{i}w_{i}p_{i}^{T}q_{i}$ of at most r(d−1)+1 matrices, where *w* is a *Kabsch-coreset* as in Definition 2.

This is done by by choosing *w* such that

(13) $$E=U^{T}\left(\sum_{i}w_{i}p_{i}^{T}q_{i}\right)V=\sum_{i}w_{i}\left(U^{T}p_{i}^{T}q_{i}V\right)$$

is a diagonal matrix. In this case, the rotation matrix of the pairs ${\left(\sqrt{w_{i}}p_{i},\sqrt{w_{i}}q_{i}\right)}_{i=1}^{n}$ and (P,Q) will be the same by Theorem 2. By letting $m_{i}=\left(U^{T}p_{i}^{T}q_{i}V\right)$ we need to have the sum $\sum_{i=1}^{n}m_{i}$ by a weighted subset of the same sum.

This vector $m_{i}$ is computed in Line 5. In Line 7 we compute a mean coreset $\left(S^{\prime },w^{\prime }\right)$ using Algorithm 2 for the *n* vectors $m_{1},\cdots ,m_{n}$. Since the mean coreset contains only the nonzero weights with their corresponding points, in Line 8 we translate the $|S^{\prime }|=O\left(d^{2}\right)$ weights in $w^{\prime }$ to the sparse vector *w*: if $s_{i}$ is the *i*th point in *S* and $s_{i}=m_{j}$, then $w_{j}=w_{i}^{\prime }$. Theorem 1 then guarantees that (13) holds as desired.

We now prove the main theorem of this section.

**Theorem** **3.** *Let* $P,Q\in R^{n\times d}$ *be a pair of matrices. Let r denote the rank of the matrix P. Then a call to the procedure* KABSCH-CORESET(P,Q) *returns a Kabsch-coreset w of sparsity at most* r(d−1)+1 *for* (P,Q) *in* $O(nd^{4})$ *time; see Definition 2.*

**Proof.**  Since $\left(S,w^{\prime }\right)$ is a mean coreset for $m_{1},\cdots ,m_{n}$ we have that *w* is a distribution of sparsity at most r(d−1)+1, such that

(14) $$E=U^{T}\left(\sum_{i}p_{i}^{T}q_{i}\right)V=U^{T}\left(\sum_{i}w_{i}p_{i}^{T}q_{i}\right)V$$

is diagonal and consists of at most *r* nonzero entries. Here $p_{i}$ and $q_{i}$ are row vectors which represent the *i*th row of *P* and *Q* respectively. Let $\left\{\sqrt{w_{i}}p_{i}|w_{i}>0\right\}$ and $\left\{\sqrt{w_{i}}q_{i}|w_{i}>0\right\}$ be the rows of $\tilde{P}$ and $\tilde{Q}$ respectively. Let $FEG^{T}$ be an SVD of $A^{T}{\tilde{P}}^{T}\tilde{Q}B$ such that det(F)det(G)=1, and let $\tilde{R}=GF^{T}$ be an optimal rotation of this pair; see Theorem 2. We need to prove that

$$\mathrm{OPT}\left(PA+\mu ,QB+\nu \right)=\mathrm{cost}\left(PA+\mu ,QB+\nu ,\tilde{R}\right).$$

We assume without loss of generality that μ=ν=0, since translating the pair of matrices does not change the optimal rotation between them [59].

By (14), $UEV^{T}$ is an SVD of ${\tilde{P}}^{T}\tilde{Q}$, and thus $A^{T}UEV^{T}B$ is an SVD of $A^{T}{\tilde{P}}^{T}\tilde{Q}B$. Replacing *P* and *Q* with $\tilde{P}A$ and $\tilde{Q}B$ respectively in Lemma 2 we have that $GD_{r}F^{T}=B^{T}VD_{r}U^{T}A$. Note that since $UDV^{T}$ is an SVD of $P^{T}Q$, we have that $A^{T}UDV^{T}B$ is an SVD of $A^{T}P^{T}QB$. Using this in Lemma 2 with PA and QB instead of *P* and *Q* respectively yields that $\tilde{R}=GF^{T}$ is an optimal rotation for the pair (PA,QB) as desired, i.e.,

$$\mathrm{OPT}\left(PA,QB\right)=\mathrm{cost}\left(PA,QB,\tilde{R}\right).$$

 □

 **Algorithm 3:** Kabsch-Coreset(P,Q) 
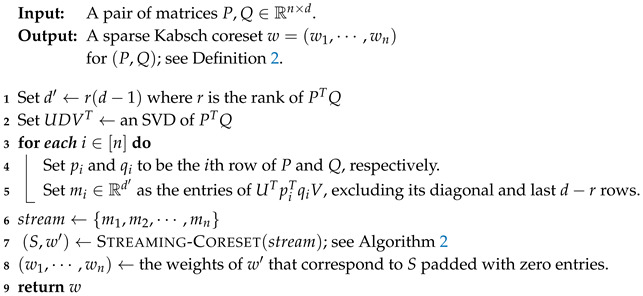


## 5. From Theory to Real Time Tracking System

While our coresets are small and optimal, they come with a price: unlike random sampling which takes sublinear time to compute (without going over the markers), computing our coreset takes the same time as solving the pose estimation problem on the same frame. Hence, we use the following pair of parallel threads.

The first thread, which we run at 1 to 3 FPS (frames per second), gets a snapshot (frame) of the currently observed markers *Q* and computes the coreset for this frame. This includes marker identification, matching problem, and then computing the actual coreset for the original set of markers *P* and the observed set *Q*. The second thread, which calculates the object’s pose, runs every frame. In our low-cost tracking system (see Section 6) it handles 30 FPS. This is by using the last computed coreset on the new frames, until the first thread computes a new coreset for a later frame. The assumption of this model is that, for frames that are close to each other in time, the translation and rotation of the observed set of markers will be similar to the translation and rotation of the set *Q* in the previous frame, up to a small error. Theorem 2 guarantees that the coreset for the first frame will still be the same for the new frame.

## 6. Experimental Results

We run the following types of experiments:

### 6.1. Synthetic Data

We constructed a set *P* of *n* randomly and uniformly sampled points in $R^{3}$, a rotation matrix $R\in R^{3\times 3}$, and a translation vector $t\in R^{d}$ from a uniform distribution. We defined Q=P·R+t and aimed to reconstruct *R* and *t* using the following methods: (i) Calculate the optimal rotation matrix and optimal translation vector from *P* and *Q*, as described in Section 4, (ii) Compute the same from the Kabsch-coreset (see Algorithm 3) of size r·(d−1)+1=7 (where r=d=3) and the Mean-coreset (see Algorithm 1) of size d+1=4, (iii) Uniform sampling of two sets of corresponding points from *P* and *Q*, one of size 7 and the second of size 4, and compute *R* and *t* from these sets.

**Figure 3:**
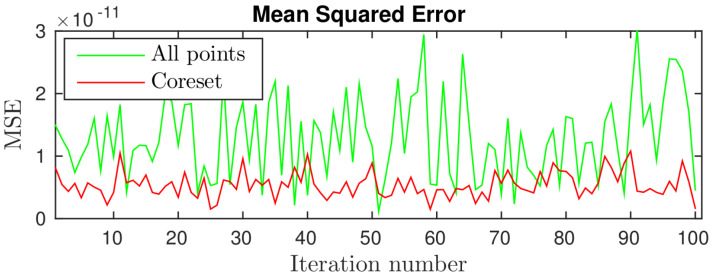
Comparing the results of methods (i) and (ii). The *X*-axis represents the number of iterations. The *Y*-axis represents the Mean Squared Errors between the two sets after applying the optimal poses obtained from each of the two methods.

**Non-noisy Data.** Here we generated data as described above for 100 iterations, where the set $P=\{p_{1},p_{2},\ldots ,p_{300}\}$ consisted of 300 randomly sampled points. Each point $p_{i}\in {\left[0,3000\right]}^{3}$, $t\in {\left[0,3000\right]}^{3}$ and *R* was randomly selected among all valid 3D rotation matrices. We then compared methods (i) and (ii) where the coreset was computed in the first iteration only and used throughout all other iterations. The results are shown in Figure 3. As proven in Section 4, the two methods yielded similar results since the data is non-noisy. Surprisingly, the coreset error in most iterations is even lower than the error of the optimal method, probably since the coreset reduces numerical errors; see the beginning of Section 3.

**Figure 4:**
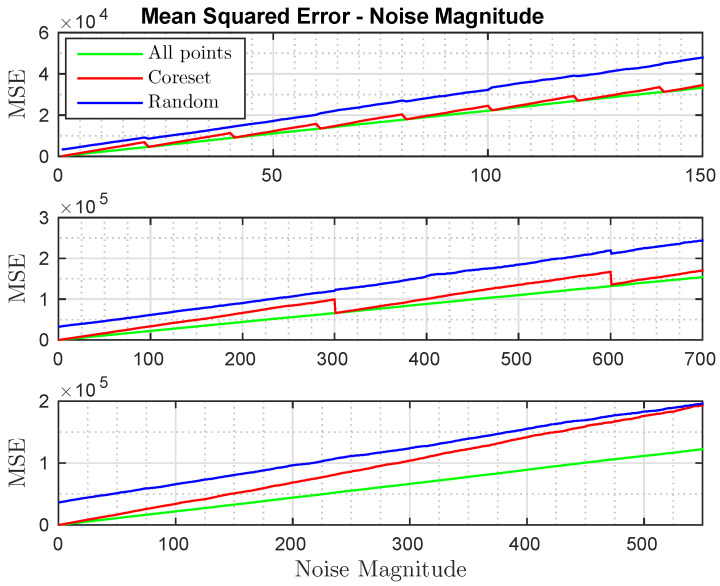
Comparing the results of methods (i), (ii), and (iii). The *X*-axis represents the noise magnitude *m*; see noise data paragraph in Section 6.1. The *Y*-axis represents the MSE between the two sets after applying the optimal poses obtained from each of the methods.

**Noisy Data.** Here our goal was to test the coresets in the presence of noise. We generated a set $P=\left\{p_{1},p_{2},\ldots ,p_{100}\right\}\in R^{100\times 3}$ of 100 randomly sampled points. Each point $p_{i}\in {\left[0,1000\right]}^{3}$, *t* is a random vector in ${\left[0,1000\right]}^{3}$ and *R* was randomly selected among all valid 3D rotation matrices. We then computed the set $Q=P\cdot R+t^{\prime }+m\cdot B$, where $B\in R^{100\times 3}$ consists of random and uniform noise in the range [0,100], *m* is the magnitude of the noise, and $t^{\prime }\in R^{100\times 3}$ is simply the concatenation of *t* 100 times. This test compares the error produced by methods (i)–(iii) while increasing the value of *m* for multiple iterations. The coreset was recomputed every *x* iterations and the random points were also resampled every *x* iterations, where *x* is the calculation cycle. The results are shown in Figure 4; the first graph shows the results for x=20, the second graph shows the results for x=300, and the third graph shows the results for x=∞ (i.e., computed only once). The results show a steady increase in the error of method (iii). Our coreset’s error steadily increases until a new coreset is recalculated; at that point the coreset error realigned with the error of method (i) as expected, resulting in stiff decreases that are seen in the graphs. Moreover, the coreset error converges to the error of the random sampling in the third graph (as expected) since the coreset is not recomputed while the noise magnitude becomes larger; in this case the coreset points do not outperform a random sample of the points.

**Figure 5:**
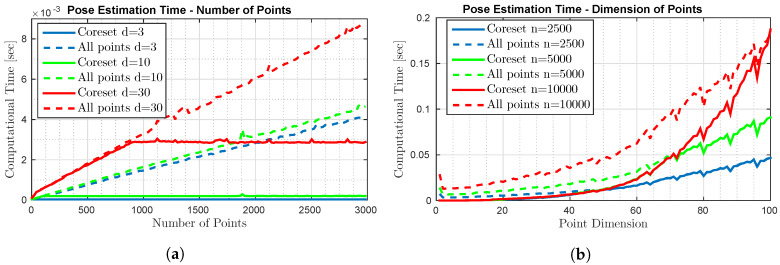
Time comparison between calculating the orientation of *n* points of dimension *d* given a previously calculated coreset versus using all *n* points.

**Running Time.** To evaluate the running time of our algorithms, we apply them on random data using a laptop with an Intel Core i7-4710HQ CPU @ 2.50GHz processor. We compared the calculation time of the pose estimation on a coreset vs. the full set. This test consists of two cases: (a) Using an increasing number of points while maintaining a constant dimension, (b) Using a constant number of points of different dimensions. The results are shown in Figure 5a,b respectively. The test corresponds to the first row of Table 1. Figure 5a shows that when the coreset size of Algorithm 3 is larger than the number *n* of points, the computation time is roughly identical, and as *n* reaches beyond $dr=O(d^{2})$, the computation time using the full set of points continues to grow linearly with *n* ($O(nd^{2})$), while the computation time using the coreset, which is dominated by the computation of the optimal rotation, ceases to increase since it is independent of *n* ($d^{3}r$ = $O(d^{4})$). Figure 5b shows that the coreset indeed yields smaller computation times compared to the full set of points when the dimension $d<\sqrt{n}$, and both yield roughly the same computation time as *d* reaches $\sqrt{n}$ and beyond.

### 6.2. IoTracker: A Multicamera Wireless Low-Cost Tracking System

We developed a wireless and low-cost home-made indoor tracking system (<$100) based on web-cams, IoT mini-computers (hence the name IoTracker), and the algorithms in this paper to compensate the weak hardware; see demonstration video in [30]. The system consists of distributed “client nodes” (one or more) and one “server node”. Each client node contains two components: (A) a mini-computer, Odroid U3 (<$30) and (B) a pair of standard web-cams (SONY PSEye, <$5). The server node consists only of a mini-computer. The server node runs the two threads discussed in Section 5.

#### Autonomous Quadcopter

We used our tracking system to compute the 6DoF of the quadcopter and send control commands accordingly after reverse engineering its communication protocol. We compared the orientation error of the quadcopter using our coreset as compared to uniform sampling of the IR or visual markers on the quadcopter.

In both tests, the coreset was computed every *x* frames, the random points were also sampled every *x* frames, where *x* is the calculation cycle time. The chosen weighted points were used for the next *x* frames, and then a new Kabsch-Coreset of size r(d−1)+1=5 was computed by Algorithm 3, where d=3 and r=2 as the features on the quadcopter are roughly in a planar configuration.

See Section 5 and the video in [30] for demonstrations and results.

**Figure 6:**
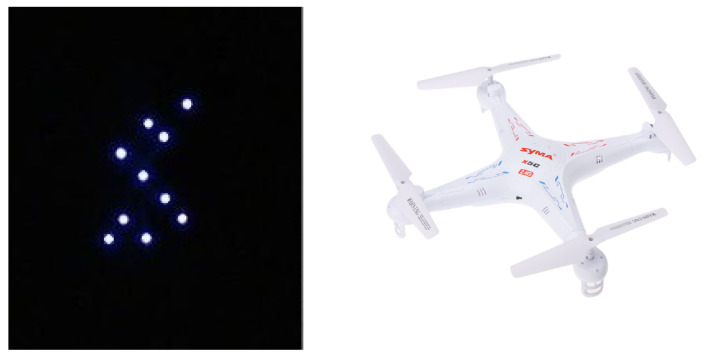
(**left**) 10 IR markers as captured by the web-camera with the IR filter. (**right**) A Syma X5C sensorless toy microquadcopter. Weight: ∼100 gr, cost: $30–$40.

**Infra-Red (IR) Tracking.** Following the common approaches used by the commercial tracking systems, we used IR markers for tracking. We placed 10 Infra-red LEDs on the quadcopter and modified the web-cams’ lenses to let only infrared spectrum rays pass, see Figure 6 (left). We could not place more than 10 LEDs on such a microquadcopter because of overweight problem and short battery life. Since the sensorless quadcopter requires a stream of at least 30 control commands per second in order to hover and not crash, we apply the Kabsch algorithm only on a selected a subset of five points. Our experiments showed that even for such small numbers, choosing the right subset is crucial for a stable system.

The system computes the 3D location of each LED using triangulation. Afterwards, it uses Algorithm 3 to compute a Kabsch-Coreset of size r(d−1)+1=5 from the 3D locations, where d=3 and r=2 as the features on the quadcopter are roughly in a planar configuration and samples a random subset (“RANSAC”) of the same size. The ground truth in this test was obtained from the OptiTrack system. The control of the quadcopter based on its positioning was done using a simple PID controller.

**Figure 7:**
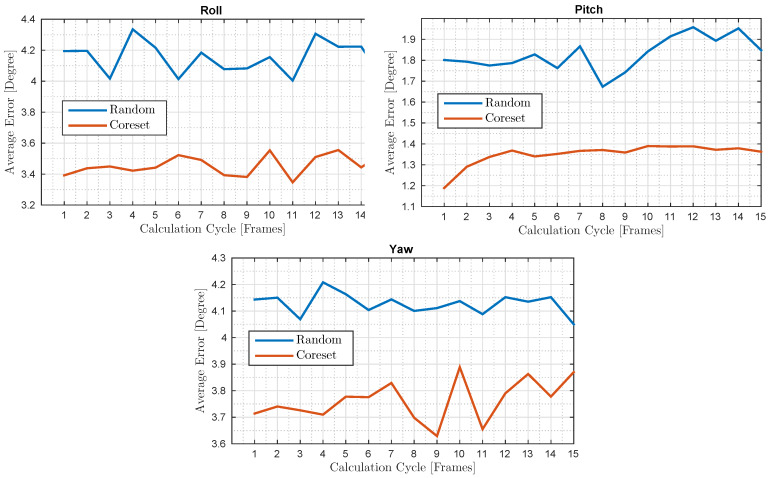
IR tracking test: For every calculation cycle (*X*-axis), we compare between the coreset average error and the uniform random sampling average error. The *Y*-axis shows the whole test average error for each calculation cycle.

For different calculation cycles, we computed the average error throughout the whole test, which consisted of roughly 4500 frames. The results are shown in Figure 7.

**RGB Tracking.** To test larger sets of points, we used our tracking system to track visual features (RGB images). We placed a simple planar pattern on a quadcopter; see Figure 1. Due to the time complexity of extracting visual features, we also placed few IR reflective markers and used the OptiTrack motion capture system to perform an autonomous hover with the quadcopter, whilst two other 2D grayscale cameras mounted above the quadcopter collected and tracked visual features from the pattern using SIFT feature detector; see submitted video. The matching between the SIFT features in both images has some mismatches. This is discussed at the end of Section 1. Given 2D coordinates of the extracted visual features from two cameras, we were able to compute the 3D location of each detected feature using triangulation. As in the IR markers test, a Kabsch-Coreset of size 5 was computed, alongside a random sample of the same size; see Figure 1. The quadcopter’s orientation was then estimated by computing the optimal rotation matrix, using the Kabsch algorithm, on both the coreset points and the random sampled points. The ground truth in this test was obtained using the Kabsch algorithm on all the points in the current frame.

**Figure 8:**
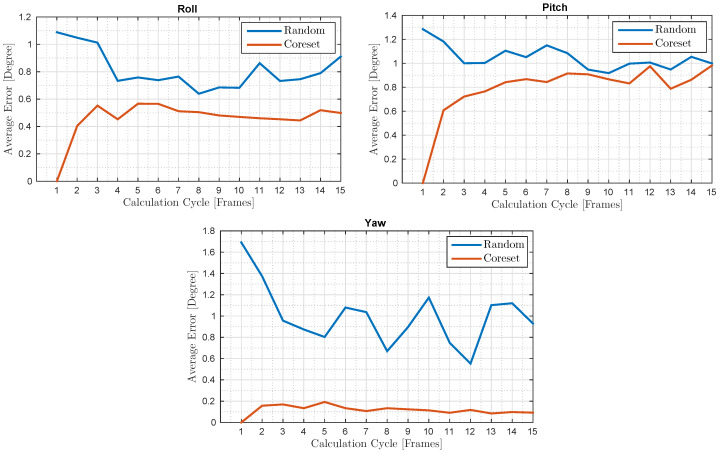
RGB Tracking test: For every calculation cycle (*X*-axis), we compare between the coreset average error and the uniform random sampling average error. The *Y*-axis shows the whole test (3000 frames) average error for each calculation cycle.

For different calculation cycles, we computed the average error throughout the whole test, which consisted of ∼3000 frames, as shown in Figure 8. The number of detected SIFT features in each frame was 60–100, though most of the features did not last for more than 15 consequent frames; therefore, we tested the coreset with calculation cycles in the range 1 to 15. The average errors were smaller than the average errors in the previous test due to the inaccurate 3D estimation using low-cost hardware in the previous test, e.g., $5 web-cams as compared to OptiTrack’s $1000 cameras and due to the difference between the ground truth measurements in the two tests.

## 7. Conclusions

We demonstrated how coresets that are usually used for solving problems in machine learning or computational geometry can also turn theorems into real-time systems. We suggested new coresets of constant size for kinematic data points in three-dimensional space. This enabled us to compute the Kabsch algorithm in real-time on slow devices by running them on the coreset, while getting provably exactly the same results. In the companion video [30] we demonstrate the first low-cost wireless tracking system that uses coresets and turns a toy quadcopter into a “Guardian Angel“ that leads guests to their desired location.

Open problems include extending our coresets for handling outliers, matching between frames, different cost functions and inputs, and multiple rigid bodies.

## Figures and Tables

**Table 1:** Time comparison. All the numbers written in the table are in *O* notation and represent time complexity.

	Without Coreset |P|=n,|Q|=n	Using Coreset |P|=rd
With matching, without rotation	$nd^{2}$	|Q|=rd $d^{3}r$
Without matching, with rotation	$n^{2.5}\log\left(n\right)$	|Q|=n $n^{1.5}dr\log\left(n\right)$
Noisy matching	$nd^{2}\cdot \left(n!\right)$	|Q|=rd (dr)!
